# Near–Real-Time Clinical Trial Accrual Dashboard in a National Cancer Institute–Designated Cancer Center: Mixed Methods Implementation Study

**DOI:** 10.2196/82920

**Published:** 2026-06-02

**Authors:** Sam Pepper, Isuru Ratnayake, Mohammod Mahmudur Rahman, Md Robiul Islam Talukder, Md Saiful Islam Saif, Olivia Rippee, Ananda Jayawardhana, Tara Lin, Natalie Streeter, Byron Gajewski, Jo Wick, Matthew S Mayo, Dinesh Pal Mudaranthakam

**Affiliations:** 1 University of Kansas Medical Center Kansas City, KS United States; 2 The University of Kansas Cancer Center Kansas City, KS United States; 3 Department of Mathematics and Physics Pittsburg State University Pittsburg, KS United States

**Keywords:** clinical trial accrual, R Shiny dashboard, data quality, implementation science, data quality, cancer center operations

## Abstract

**Background:**

Clinical trial accrual monitoring is a critical component of trial operations, influencing feasibility, timeliness, and scientific validity. Despite its importance, many National Cancer Institute–designated cancer centers continue to rely on static spreadsheets or manually generated reports that provide delayed and incomplete insight for study teams. These limitations hinder timely identification of recruitment challenges, reduce transparency across stakeholders, and constrain proactive operational decision‑making. Scalable, institution‑wide systems that support near–real‑time accrual oversight remain uncommon in academic settings.

**Objective:**

This study aimed to design, implement, and operationalize a near–real‑time clinical trial accrual dashboard within a National Cancer Institute–designated cancer center, and to evaluate its use, adoption, and early operational impact on accrual monitoring workflows.

**Methods:**

We developed an enterprise accrual dashboard integrating daily extracts from the institutional clinical trial management system with automated data quality validation and time series accrual forecasting. The system supported multiple stakeholder roles through role‑based access and was deployed within existing governance and oversight workflows. Implementation and evaluation were guided by the reach, effectiveness, adoption, implementation, and maintenance (RE-AIM) framework and the Guidelines and Checklist for the Reporting on Digital Health Implementations framework, with system logs used to assess reach and adoption, and qualitative operational feedback used to characterize use in practice.

**Implementation (Results):**

From May 2022 to May 2026, the dashboard was used institution wide for monitoring therapeutic and nontherapeutic trials, logging 1605 unique user sessions across investigators, coordinators, data managers, and leaders. Automated daily validation enabled earlier detection and correction of missing or invalid accrual data, improving the reliability of institutional reporting over time. The dashboard replaced manual monthly reports with continuous monitoring and was incorporated into routine operational and leadership reviews, enabling earlier identification of lagging studies and more timely discussions regarding resource allocation and recruitment strategies. Accrual forecasting models demonstrated stable in sample and out of sample performance and were used to support anticipatory planning rather than prescriptive decision making. Initial increases in data correction workload and user resistance decreased as workflows matured and data transparency became normalized.

**Conclusions:**

Implementation of a near–real‑time accrual dashboard within an academic cancer center is feasible and sustainable and can meaningfully improve accrual oversight by strengthening data quality, transparency, and integration into routine workflows. The primary value of such systems lies not only in visualization or analytics but in their ability to shift accrual monitoring from retrospective reporting to proactive, institution‑wide operational review. With appropriate governance and clinical trial management system infrastructure, similar dashboards are transferable to other research centers seeking to modernize trial oversight.

## Introduction

The National Cancer Institute cancer centers throughout the United States deal with enormous amounts of data stemming from their portfolios of clinical trials. Extracting meaningful insights from this data can be a challenging task, given its scale, complexity, and the involvement of multiple stakeholders, including clinicians, researchers, and administrators [1]. Therefore, accurate reporting from these data necessitates robust standard operating procedures (SOPs), comprehensive training, and a solid infrastructure [2-4]. In this context, infrastructure refers to the data processing and dissemination of clinical trial accrual information [4]. The lack of a unified, well-structured infrastructure for data handling can result in inaccurate reporting and flawed conclusions, ultimately leading to considerable waste of time and financial resources. Additionally, a practical understanding of patient participation in clinical trials is crucial for advancing cancer research and maximizing patient outcomes [5,6].

The timely accrual of eligible participants is the prerequisite for the success of these trials, as insufficient recruitment remains the leading cause of premature study termination for both oncology and nononcology studies [7,8]. A study analyzing trials registered on ClinicalTrials.gov found that 12% (905/7646) were terminated early, with inadequate participant accrual being the primary reason, responsible for 57% (350/619) of these terminations [9]. In oncology, the situation is also difficult; research indicates that 50% of phase III cancer trials close due to insufficient accrual [10]. Termination of clinical trials due to inadequate accrual can have a significant adverse impact on sponsors’ and participating sites’ financial and other resources. Trials that are delayed or terminated early hinder the development of effective therapies for patients who urgently need them [7]. Therefore, understanding historical accrual trends and forecasting future accrual trends is essential for the success of clinical trials.

Clinical trial programs at National Cancer Institute–designated cancer centers generate large volumes of complex operational data, and the accuracy of this information depends heavily on robust data infrastructure, SOPs, and strong governance frameworks. Foundational guidance on SOP development and informatics workflows emphasizes that without consistent processes, institutions risk inaccurate reporting and inefficient resource use, ultimately affecting trial quality and outcomes [2-4]. Ensuring data integrity remains essential not only for scientific validity but also for ethical and operational efficiency, particularly as patient participation plays a central role in advancing cancer research and improving outcomes [5,6].

Timely participant accrual remains one of the most significant barriers to successful trial completion. Insufficient recruitment remains the leading cause of early trial termination across oncology and nononcology trials, with multiple studies documenting high rates of closure due to inadequate enrollment [7-10]. These challenges have substantial financial and operational implications, delaying access to potentially beneficial therapies and hindering progress in clinical research.

To address these issues, many institutions have developed tools to visualize accrual trends, improve operational insight, and support recruitment‑related decision‑making. For example, the University of Rochester Medical Center and the University of Arizona have both created data dashboards to track patient accrual and study performance, while Baylor College of Medicine and Columbia University have deployed similar systems tailored to their institutional needs [11-14]. These dashboards illustrate a broader shift toward interactive, data‑driven monitoring platforms that allow stakeholders to examine trends, identify emerging barriers, and facilitate earlier intervention.

Within this evolving landscape, the University of Kansas Cancer Center’s (KUCC) near–real‑time accrual dashboard was designed to unify these core functions: streamlined data extraction, data quality monitoring, flexible visualization, and forecasting into a single operational tool. By integrating these capabilities, the dashboard supports more consistent oversight of accrual performance and aligns with contemporary approaches to trial management that prioritize accuracy, efficiency, and proactive decision‑making.

## Methods

### Data Sources and Management

Clinical trial operational data were extracted daily from the WCG Velos clinical trial management system (CTMS), which stores information in an Oracle relational database. Automated structured query language queries retrieved patient‑level and study‑level metrics without interrupting CTMS operations. Extracted data underwent standardization and cleaning through R scripts using dplyr, janitor, zipcodeR, and lubridate, correcting formatting inconsistencies, invalid zip codes, and improperly structured dates. Records with missing or incorrect geographic identifiers were automatically flagged and emailed to the KUCC clinical trials office for timely correction. Rurality designations were assigned using 2023 Rural-Urban Continuum Codes and 2010 Rural-Urban Commuting Area codes, and cleaned data were saved as encrypted RData files prior to secure upload.

### Dashboard Architecture and Data Integration

The dashboard was implemented using the *flexdashboard* and *shiny* frameworks in R (Posit Software). Each dashboard tab was constructed with reactive filters and visualization modules (eg, summary tables, pivot tables, line graphs, bar charts, and Plotly‑based interactive graphics). Subsetting functions responded to user-selected filters (eg, study characteristics, demographics, and date ranges), and visual outputs were dynamically regenerated. Primary packages included *flexdashboard*, *shinyWidgets*, *DT*, *Plotly*, and *rpivotTable*. Additional visualization of dashboard functionality, including user interface components, accrual summaries, timeline views, forecasting outputs, and data exploration features, is provided in Multimedia Appendix 1 (Figures S1–S7) to further illustrate system design and operational capabilities.

### System Infrastructure and Security

The dashboard was hosted on a Red Hat Enterprise Linux server running Shiny server, with https encryption provided through an Nginx reverse proxy. User sessions initiated secure connections to the Microsoft SQL Server housing encrypted RData files. Access was restricted to KUCC personnel, and geographic identifiers were processed only for aggregate reporting to prevent individual reidentification. The system operated within institutional governance frameworks and complied with institutional review board (IRB) requirements.

### Implementation and Evaluation Framework

Dashboard deployment and evaluation followed the reach, effectiveness, adoption implementation, and maintenance (RE-AIM) framework, assessing reach, adoption, effectiveness, implementation fidelity, and maintenance. System‑generated logs quantified user engagement across roles and sites. Effectiveness was evaluated based on operational outcomes such as detection of missing data, accrual monitoring utility, and forecasting support. Implementation fidelity was assessed through governance processes, validation rules, and role‑based training procedures. Adoption and maintenance were tracked through sustained use over time and workflow integration.

### Ethical Considerations and Regulatory Compliance

This project was reviewed and approved by the University of Kansas Medical Center Institutional Review Board as non–human subjects research due to its reliance on cumulative, deidentified operational data. All data handling complied with institutional privacy policies, and the dashboard operated on secure, access‑restricted servers using anonymized datasets.

## Implementation (Results)

### Data Sources and Management: Operational Outcomes

Daily automated validation procedures consistently identified missing or invalid data entries, including zip codes, dates, and demographic fields, leading to prompt correction by the clinical trials office. These near–real‑time quality checks strengthened data integrity feeding the dashboard, reduced downstream discrepancies, and improved the reliability of accrual reporting.

Consistent with the Guidelines and Checklist for the Reporting on Digital Health Implementations reporting guidelines, the results describe key implementation outcomes, including system reach, adoption across user roles, operational use, data quality processes, and early maintenance indicators within routine cancer center workflows.

### Implementation Experience and Workflow Impact

Implementation of the accrual dashboard led to several operational insights that extended beyond its technical features. Automated daily data validation revealed recurrent upstream data quality issues particularly missing demographic fields and delayed study status updates which initially increased correction workload for clinical trials office staff. However, this early surge was followed by improved consistency in data entry practices as coordinators became more aware of downstream visibility. Across roles, users reported that near–real‑time access to accrual trends reduced dependence on manually prepared monthly reports and allowed earlier identification of lagging studies. Leadership users particularly valued longitudinal and forecast views for program‑level oversight, while study teams used the dashboard to support operational discussions around recruitment barriers and resource allocation.

### System Infrastructure and Security: Observed Performance

System logs indicated 1605 unique user sessions between May 2022 and May 2026, demonstrating sustained adoption by investigators, coordinators, data managers, and institutional leadership. Role‑based access functioned as intended, and the secure hosting environment supported stable, encrypted, and uninterrupted access.

### Implementation and Evaluation Outcomes (RE-AIM)

Implementation and evaluation outcomes were assessed using the RE-AIM framework, demonstrating strong performance across all its dimensions (Textbox 1, Tables 1 and 2).

**The reach, effectiveness, adoption implementation, and maintenance (RE-AIM) framework.**

- Maintenance: continuous use through 2025 with periodic feature enhancements and integration into standard oversight processes.

**Table 1:** Reach and adoption information.

Year	Number of sessions (N=1605), n (%)	Cumulative sessions, n	Implementation insight
2022	284 (17.7)	284	Initial rollout and training phase
2023	506 (31.5)	790	Rapid uptake across coordinators, data managers, and leadership
2024	323 (20.1)	1113	Sustained maintenance and integration into daily workflows
2025	389 (24.2)	1502	Continued growth with implementation of predictive enhancements
2026	103 (6.4)	1605	Ongoing use (Q1 only) and addition of leaderboards for DWG disease working group and PIsprincipal investigators

**Table 2:** Effectiveness, implementation, and maintenance information.

Stakeholder group	Key use cases	Enhancements leveraged	Demonstrated value to cancer center operations
Study coordinators	Real-time accrual monitoring, recruitment forecasting, and EDC^a^ data validation	Accrual prediction and data cleaning	Accelerated enrollment decisions; reduced manual tracking time; proactive identification of recruitment barriers
Data managers	EDC validation, data cleaning, discrepancy flagging, and report exports	Ad hoc reporting and data cleaning	Improved data quality and integrity; faster resolution of data issues
Project managers	Milestone tracking, resource allocation, and cross-trial reporting	Accrual prediction and data cleaning	Efficient study management
Leadership	Strategic planning, road-map development, and accrual forecasting	Accrual prediction, disease working group DWG and principal investigatoror PI leaderboard, and forecast modeling	Data-driven decision-making at the center level
Physicians and or investigators	Enrollment trend visualization and feasibility assessment	Disease working group and principal investigator DWG/PI leaderboard, accrual prediction, and forecast modeling	Enhanced investigator engagement; real-time performance benchmarking; better trial selection and patient allocation

^a^EDC: electronic data capture.

### Ethics and Compliance Outcomes

Throughout the study period, all dashboard operations complied with institutional privacy protections and IRB guidelines. No identifiable participant‑level information was displayed, and all outputs were aggregated to prevent reidentification.

## Discussion

### Key Findings

The KUCC accrual dashboard aligns with a growing body of work emphasizing the need for improved accrual efficiency, real‑time data visibility, and proactive trial management. Numerous studies have documented persistent challenges in recruitment, particularly in oncology where delays, early closures, and insufficient accrual continue to undermine trial success [15-19]. These barriers underscore the need for tools that enable early detection of accrual shortfalls, improved operational oversight, and informed decision‑making.

The dashboard’s forecasting capabilities, based on autoregressive integrated moving average, error trend, and seasonality, and Prophet models, directly support this need by enabling more accurate projections of future accrual performance. Similar analytic frameworks have been used to evaluate prospective recruitment, inform trial continuation decisions, and reduce preventable early terminations [18,19]. More broadly, research highlights how forecasting methods can reduce operational uncertainty, optimize resource allocation, and guide intervention strategies to maintain enrollment momentum.

Data quality is another critical component of clinical trial oversight. Prior work has shown that inconsistent, incomplete, or erroneous data compromise trial integrity, delay analyses, and introduce risk across operational stages [20-23]. By implementing automated daily validation checks, the KUCC system reflects established best practices for maintaining clean, reliable data pipelines in clinical research environments. This continuous auditing approach helps correct issues earlier in the data life cycle and enhances the accuracy of downstream accrual reporting.

Efforts to improve equitable participation in clinical trials have further highlighted the importance of monitoring demographic patterns, addressing disparities, and ensuring representation across diverse patient groups [24-26]. By enabling users to assess accrual by demographic variables and geographic classifications, the dashboard supports targeted strategies to address underrepresentation and supports institutional goals related to diversity in clinical research.

Finally, the dashboard aligns with implementation frameworks, such as iCHECK‑DH, which emphasize system usability, data governance, stakeholder involvement, and long-term sustainability [1,27] (see Table S2 in Multimedia Appendix 2). Its modular Shiny‑based architecture, centralized data processing, and role‑based access control support scalable, secure, and maintainable integration into routine workflows. As clinical research grows more complex, such systems are increasingly essential for reducing delays, improving transparency, and ensuring high‑quality operational execution.

Overall, the KUCC accrual dashboard represents a modern, practical solution for improving accrual oversight, enhancing data quality, and supporting responsive, analytics‑driven trial management. Its capabilities align with contemporary expectations for digital health tools and position the institution for continued improvements in trial efficiency and performance.

### Transferability and Scalability Considerations

Adoption of this accrual dashboard at other institutions would require several foundational elements. First, centers must have access to a well-established CTMS capable of generating structured, study-level and participant-level accrual data through automated extracts. Although this implementation leveraged WCG Velos, the dashboard architecture is modular and can be adapted to other CTMS platforms (eg, OnCore or locally developed systems) provided that standardized accrual variables and metadata are available. Second, robust institutional data governance is essential, including IRB oversight where applicable, defined data stewardship roles, and policies governing access, security, and secondary use of operational trial data. Third, successful deployment depends on dedicated staffing resources, including informaticians or data engineers to manage data pipelines, biostatistics or analytics personnel to maintain forecasting models, and clinical research operations staff to respond to data quality flags and incorporate dashboard insights into routine workflows. Finally, sustainability requires ongoing maintenance, such as scheduled data refreshes, validation rules to ensure data integrity, user support, and periodic system enhancements aligned with evolving institutional needs. While the technical infrastructure required is modest relative to enterprise commercial solutions, effective adoption depends on alignment across informatics, governance, and operational leadership.

### Implementation Lessons Learned

Several facilitators and barriers were identified during implementation of the accrual dashboard. Key facilitators included strong institutional leadership support, integration with an existing CTMS infrastructure, and alignment with established governance and reporting workflows. Automated quality control processes were particularly influential, as they shifted data validation earlier in the data life cycle and promoted shared accountability for accrual data accuracy.

Barriers included variability in historical data quality, differences in study‑level data entry practices, and initial resistance related to increased transparency of accrual performance. Some users also experienced short‑term workflow disruption as missing or inconsistent data were surfaced more frequently. Over time, these challenges diminished as teams adapted workflows and incorporated dashboard review into routine operational meetings.

Overall, the primary workflow impact observed was a transition from retrospective, static accrual reporting to more proactive and continuous monitoring. This shift enabled earlier detection of recruitment shortfalls, improved communication between operational and leadership teams, and more timely corrective actions. These findings highlight that the value of accrual dashboards lies not only in visualization capabilities but in how they reshape institutional workflows, accountability, and decision‑making processes. Key facilitators, barriers, workflow impacts, and actionable implementation recommendations are summarized in Table S2 in Multimedia Appendix 3 to guide organizations considering similar accrual monitoring solutions.

### Implementation Lessons Learned and Recommendations for Practice

Implementation of the near–real‑time accrual dashboard generated several transferable lessons for academic cancer centers and clinical research organizations, underscoring that successful digital health tools depend as much on governance, workflow integration, and change management as on technical design.

Key facilitators included strong leadership support, early alignment with the existing CTMS infrastructure, and embedding dashboard review into routine operational processes. Automated daily data quality checks were particularly impactful, shifting data validation earlier in the data life cycle and fostering shared accountability among study teams, data managers, and leadership. Role‑based access and intuitive visualization further supported broad adoption with minimal training.

Identified barriers included variable historical data quality, inconsistent study‑level data entry practices, and short-term increases in workload as data discrepancies became more visible. Increased transparency also generated initial resistance among some users; however, these challenges diminished as workflows matured, upstream data practices improved, and dashboard use became normalized in standing meetings.

The primary workflow impact was a shift from retrospective, static reporting to continuous, proactive accrual monitoring, enabling earlier identification of recruitment challenges and more timely, data‑informed decision‑making at both study and program levels. Overall, the dashboard’s value stemmed not only from its analytic capabilities but from its role in reshaping institutional oversight and accountability.

### Strengths and Limitations

The first limitation is that the dashboard within the CTMS platform provides real-time information, whereas the KUCC accrual dashboard updates its information daily. However, a maximum 24-hour delay is not a significant concern for most research projects. A major strength of the KUCC accrual dashboard is that it provides more summary and visual information than the CTMS built-in reports. Additionally, it performs quality control checks on the exported data, unlike the other reports.

Another limitation of this work is the absence of formal quantitative outcome metrics directly attributing changes in accrual or data quality to dashboard implementation. Given the dashboard’s role as an operational and decision-support tool, isolating its independent effect from concurrent process improvements is challenging. Future studies will focus on defining and evaluating standardized metrics to quantify their impact on accrual efficiency, data completeness, and reporting timeliness.

### Conclusions

The KUCC accrual dashboard represents a scalable, implementation‑ready tool for near–real‑time monitoring of clinical trial accrual within an academic cancer center. In its current form, the dashboard demonstrably supports improved data transparency, routine data quality monitoring, historical trend analysis, and accrual forecasting, and it has been successfully integrated into operational workflows for institutional oversight. Its primary contribution lies in facilitating earlier identification of accrual and data quality issues and enabling more proactive, institution‑wide review rather than in directly measuring downstream effects on accrual performance or trial outcomes.

The dashboard continues to evolve, and future enhancements, such as geospatial visualization of accrual patterns and integration with additional data sources, including electronic health records, may further expand its analytic scope. However, the impact of these features on accrual efficiency, trial completion, and equity remains prospective and will require targeted, longitudinal evaluation. With appropriate CTMS infrastructure, governance, and staffing, similar dashboards could be adapted at other research institutions to support operational trial management within local constraints.

## Data Availability

The datasets generated during and/or analyzed during this study are not publicly available due to Health Insurance Portability and Accountability Act restrictions. However, a deidentified dataset is available from the corresponding author on reasonable request.

## Appendix

Multimedia Appendix 1 Implementation lessons learned and recommendations for practice.

Multimedia Appendix 2 Mapping of manuscript content to iCHECK-DH (Implementation Reporting Guidelines for Digital Health Interventions) domains.

Multimedia Appendix 3 Dashboard architecture and user interface details.

## Abbreviations

CTMS: clinical trial management system
IRB: institutional review board
KUCC: University of Kansas Cancer Center
SOP: standard operating procedure
RE-AIM: reach, effectiveness, adoption, implementation, and maintenance
