# Supplementary material for: Near–Real-Time Clinical Trial Accrual Dashboard in a National Cancer Institute–Designated Cancer Center: Mixed Methods Implementation Study
Source: JMIR Med Inform. 2026 Jun 2;14:e82920. doi: 10.2196/82920 (PMC13273207; doi:10.2196/82920)
Supplement: Multimedia Appendix 1 [file medinform_v14i1e82920_app1.docx]

Dashboard Architecture and User Interface Details:

Overall Summary Output: The “Overall Summary” tab (Figure 1) provided interactive, filter‑driven tables summarizing accrual across key study characteristics and participant demographics. Users could view distributions across sex, race, ethnicity, rurality, age groups, disease group, and site characteristics.


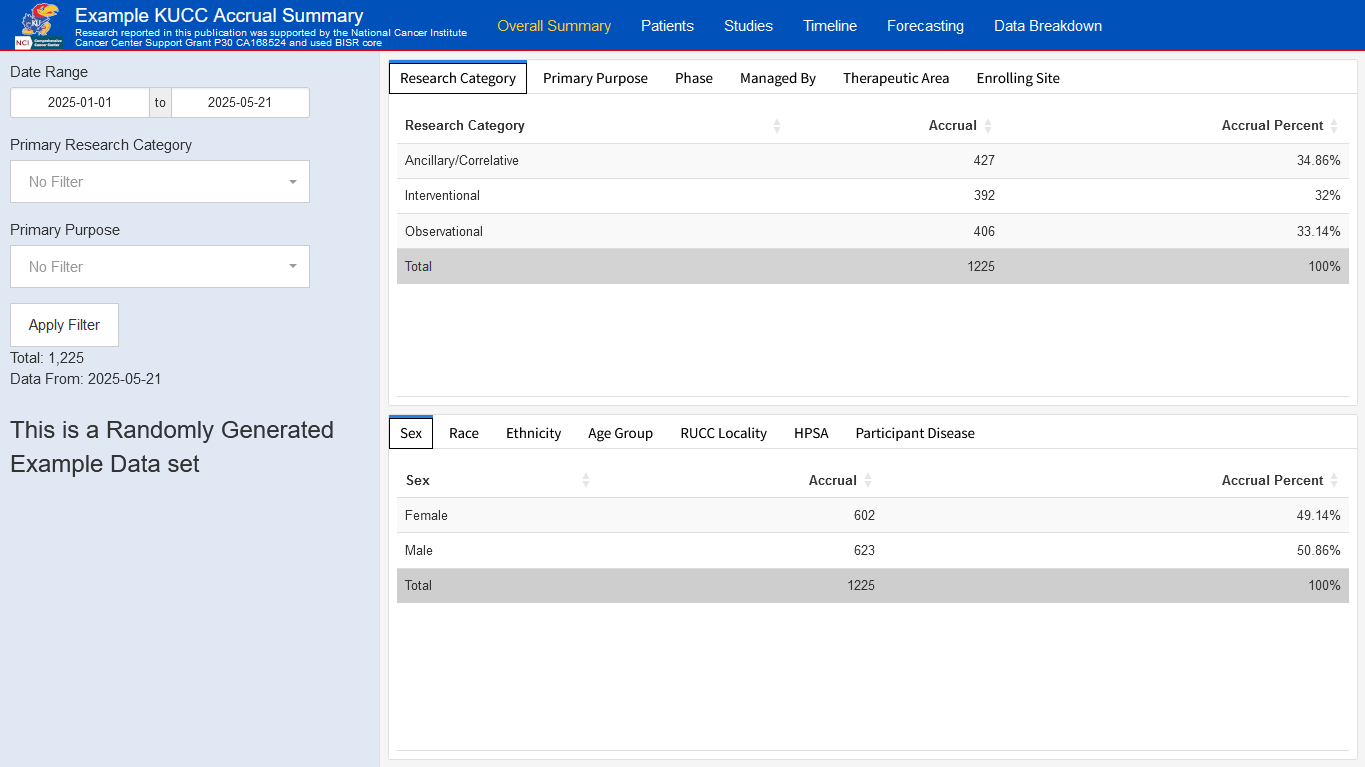


*Figure S1: Accrual Dashboard - Overall Summary Tab*


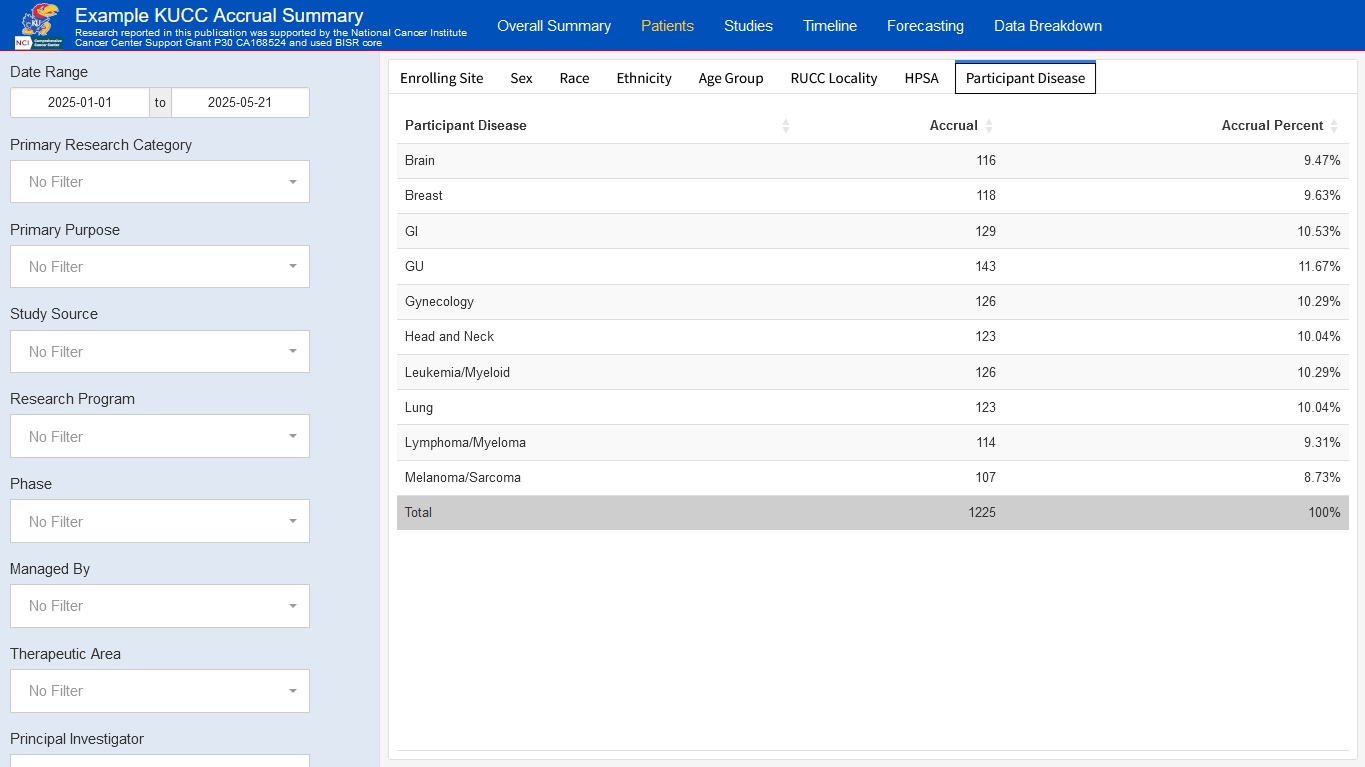

*Figure S2****:*** *Accrual Dashboard: Patients Tab*

Study‑Level Summaries: The “Studies” tab (Multimedia Appendix Figure S3) offered analogous visualizations at the study level, complementing the patient‑level summaries by allowing users to explore key study metadata and operational attributes.


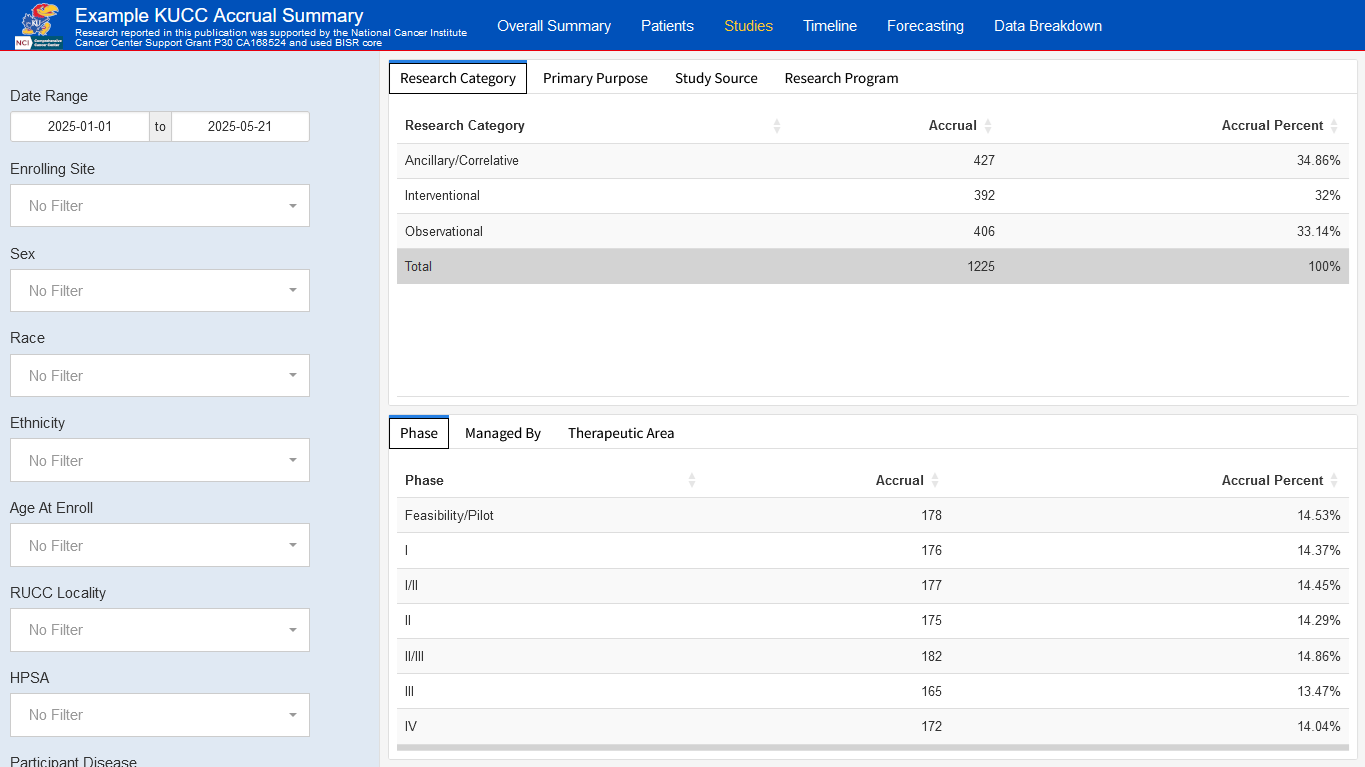


*Figure S3: Accrual Dashboard: Studies Tab*


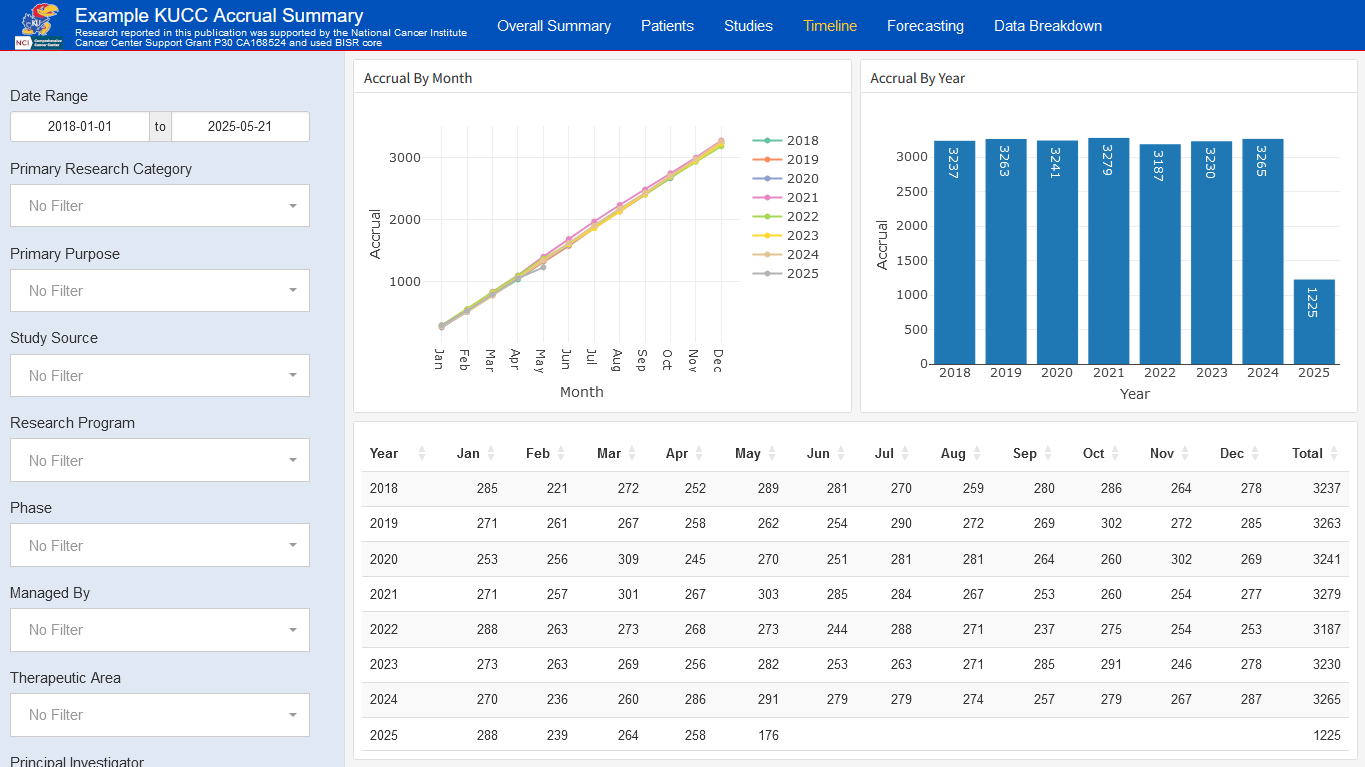


*Figure S4:* Accrual Dashboard: Timeline Tab

Accrual Trends and Timeline Visualization: The “Timeline” tab enabled users to examine longitudinal accrual patterns both cumulative and monthly allowing rapid identification of recruitment trends and deviations from expected trajectories.

Forecasting Performance and Model Evaluation: The “Forecasting” tab (Multimedia Appendix Figure S5) delivered model‑based accrual projections using autoregressive integrated moving average (ARIMA) models, exponential smoothing state space (ETS) models, and Prophet approaches. root mean squared error (RMSE) and mean absolute error (MAE) metrics were calculated for both in‑sample and out‑of‑sample validation periods. Final models generated 12‑month projections with 95% prediction intervals, supporting proactive identification of anticipated shortfalls.

**
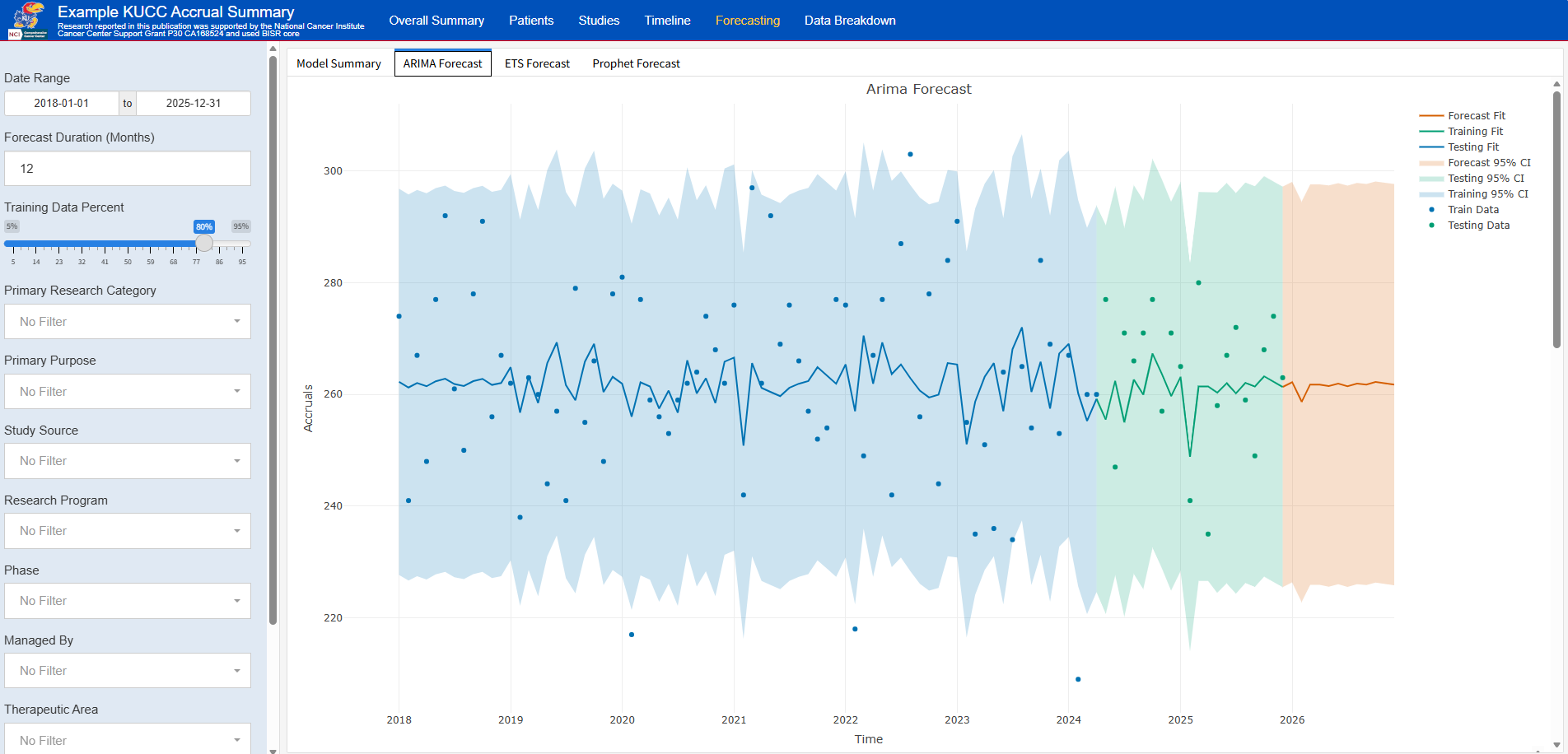
**

*Figure S5: Accrual Dashboard: Forecasting Tab*

Data Exploration and Custom Analytics: The “Data Breakdown” tab (Multimedia Appendix Figure S6) provided a drag‑and‑drop pivoting interface supporting customizable summaries and multi‑modal visualizations (heatmaps, treemaps, bar charts, line charts, etc.), enabling deeper ad hoc exploration of accrual and study characteristics

**
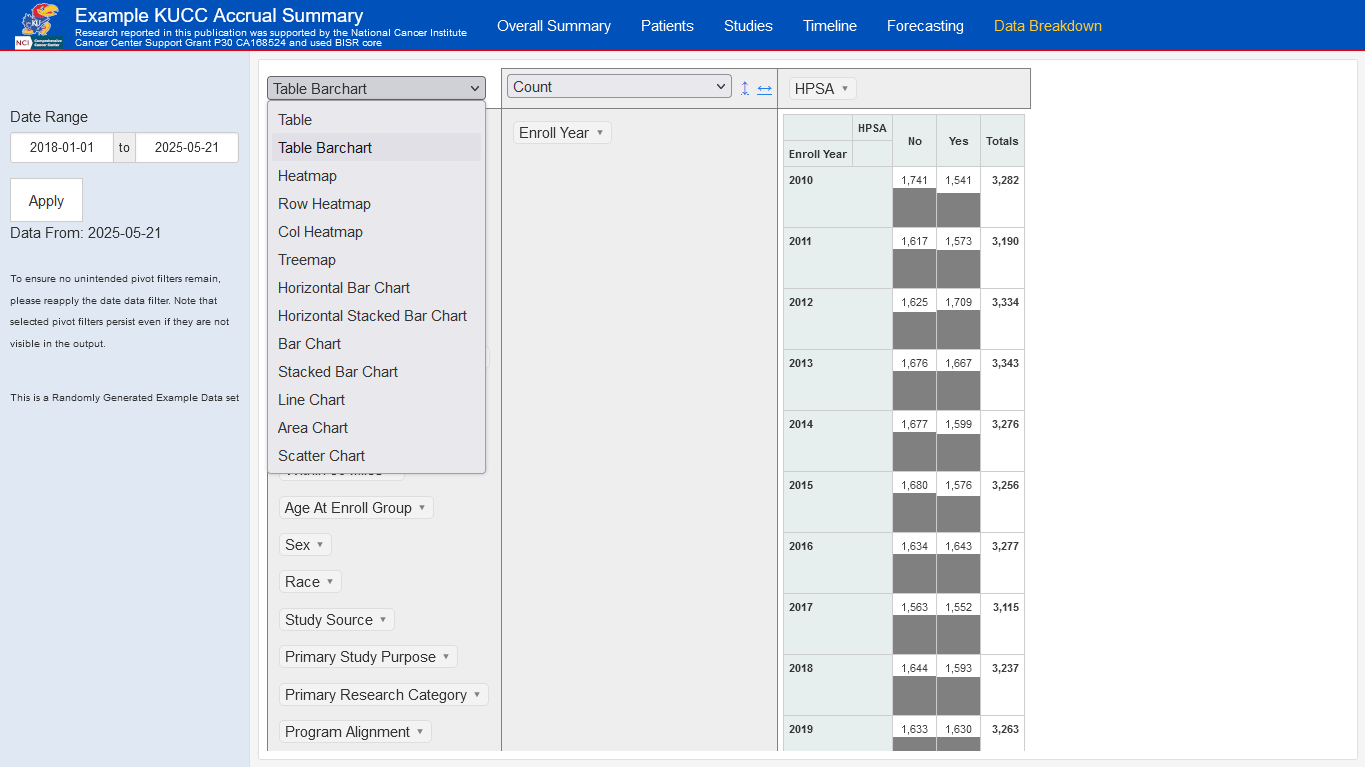
**

*Figure S6: Accrual Dashboard: Data Breakdown Tab*

The Timeline tab enables users to examine cumulative and monthly accrual trends over time (Multimedia Appendix Figure S4). The Forecasting tab provides interactive visualization of observed accruals alongside short‑term projections generated using time‑series methods (ARIMA, ETS, and Prophet), allowing users to specify date ranges and evaluate model performance using standard accuracy metrics. Selected models generate 12‑month accrual forecasts with 95% prediction intervals to support proactive planning (Multimedia Appendix Figure S5).

The Data Breakdown tab offers flexible, drag‑and‑drop data exploration through pivot tables and configurable visualizations, enabling users to generate customized summaries of accrual and study characteristics across selected variables and time periods (Multimedia Appendix Figure S6; Multimedia Appendix Figure S7).

**
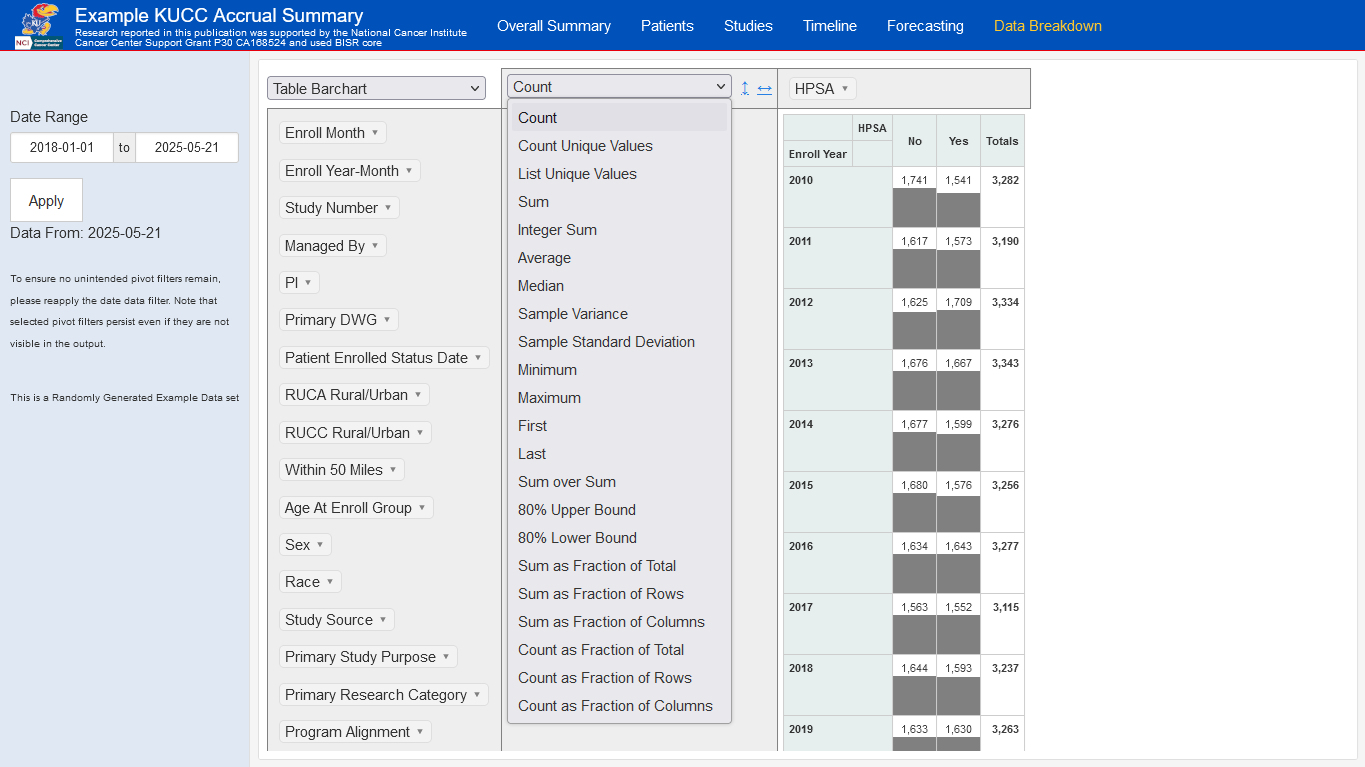
**

*Figure S7***:** Accrual Dashboard: Data Breakdown Tab
