# Supplementary material for: Near–Real-Time Clinical Trial Accrual Dashboard in a National Cancer Institute–Designated Cancer Center: Mixed Methods Implementation Study
Source: JMIR Med Inform. 2026 Jun 2;14:e82920. doi: 10.2196/82920 (PMC13273207; doi:10.2196/82920)
Supplement: Multimedia Appendix 2 [file medinform_v14i1e82920_app2.docx]

**Multimedia Appendix**

| **iCHECK-DH Domain** | **Reporting Item** | **Description of How Addressed** | **Manuscript Section(s)** |
| --- | --- | --- | --- |
| **Intervention Description** | Digital health intervention overview | Describes the purpose, scope, and core functionalities of the R Shiny–based accrual dashboard, including near–real-time data integration, visualization, and forecasting | Abstract; Introduction; Methods (Data Integration, Housing the Dashboard) |
| **Context** | Organizational and clinical setting | Describes implementation within an NCI-designated comprehensive cancer center, integration with institutional CTMS, and role within routine trial oversight workflows | Introduction; Methods (Data Management, Data Security) |
| **Stakeholders** | Intended users and roles | Identifies clinicians, principal investigators, study coordinators, data managers, statisticians, and leadership as primary user groups | Methods (Data Management; Data Integration); Results |
| **Implementation Process** | Development and deployment | Details system architecture, automated data extraction, cleaning, validation, encryption, hosting environment, and deployment strategy | Methods (Data Management; Data Integration; Housing the Dashboard) |
| **Data Governance & Security** | Privacy, security, and access control | Describes local hosting, restricted institutional access, encrypted data storage, role-based access, and aggregation to prevent re-identification | Methods (Data Security) |
| **Fidelity** | Consistency with intended use | Reports that dashboard functionality operates as designed with daily data refreshes, automated quality checks, and stable forecasting workflows | Methods; Results |
| **Reach** | Exposure to target users | Reports number of unique user sessions and breadth of use across institutional roles | Results; Discussion |
| **Adoption** | Uptake by intended users | Describes sustained use since implementation and incorporation into routine accrual monitoring by multiple stakeholder groups | Results; Discussion |
| **Effectiveness (Implementation Outcomes)** | Operational usefulness | Reports qualitative and quantitative indicators including improved transparency, data quality monitoring, accrual forecasting, and decision support | Results; Discussion |
| **Maintenance** | Sustained use over time | Describes continued use since 2022, integration into standard workflows, and ongoing updates to meet evolving needs | Results; Discussion |
| **Unintended Consequences** | Potential negative effects | Notes increased visibility of data discrepancies and short-term workload increases associated with data correction | Methods (Implementation Evaluation); Discussion |
| **Transferability / Scalability** | Applicability to other settings | Discusses modular architecture and potential adaptability to other cancer centers and CTMS platforms | Discussion; Conclusion |
| **Ethical Considerations** | Ethics and approvals | States IRB determination of non–human subjects research and compliance with institutional policies | Ethics Statement |
| **Reporting Standards** | Guideline adherence | Explicitly states adherence to iCHECK-DH implementation reporting guidelines | Methods; Results |

**Supplementary Table S1. Mapping of manuscript content to iCHECK-DH (Implementation Reporting Guidelines for Digital Health Interventions) domains.**

| **Domain** | **Key Observation** | **What Worked / What Did Not** | **Workflow Impact** | **Actionable Recommendation** |
| --- | --- | --- | --- | --- |
| Leadership & Governance | Executive sponsorship supported adoption | - Strong leadership engagement facilitated normalization - Limited early enforcement slowed uptake | Accelerated institutional alignment | Secure leadership endorsement and embed dashboard review into standing oversight meetings |
| Data Infrastructure | CTMS integration was essential | - Automated daily extracts enabled reliability - Historical data inconsistencies required remediation | Improved long‑term data quality | Conduct baseline data audits and plan for early data cleanup before launch |
| Data Quality Processes | Automated validation added value | - Early detection of missing/invalid fields - Initial increase in correction workload | Shifted QC upstream | Pair automated QC with clear ownership and response workflows |
| User Adoption | Role‑based design improved usability | - Minimal training required - Early skepticism around transparency | Sustained engagement across roles | Use role‑specific views and emphasize supportive—not punitive—use of data |
| Transparency & Culture | Increased visibility changed behavior | - Encouraged accountability - Initial resistance to comparative views | Cultural shift toward data‑driven discussions | Frame dashboards as improvement tools and socialize expectations early |
| Operational Decision‑Making | Forecasting supported proactive planning | - Enabled anticipatory interventions - Forecast uncertainty required contextual interpretation | Earlier identification of accrual risks | Pair forecasts with operational context and qualitative input from study teams |
| Maintenance & Sustainability | Continuous support was required | - Modular design enabled enhancements | Long‑term integration into workflows | Allocate dedicated informatics and analytics support for maintenance |

**Supplementary Table S2. Implementation lessons learned and recommendations for practice**
