# Supplementary material for: Near–Real-Time Clinical Trial Accrual Dashboard in a National Cancer Institute–Designated Cancer Center: Mixed Methods Implementation Study
Source: JMIR Med Inform. 2026 Jun 2;14:e82920. doi: 10.2196/82920 (PMC13273207; doi:10.2196/82920)
Supplement: Multimedia Appendix 3 [file medinform_v14i1e82920_app3.docx]

**Multimedia Appendix**

| **Domain** | **Key Observation** | **What Worked / What Did Not** | **Workflow Impact** | **Actionable Recommendation** |
| --- | --- | --- | --- | --- |
| Leadership & Governance | Executive sponsorship supported adoption | - Strong leadership engagement facilitated normalization - Limited early enforcement slowed uptake | Accelerated institutional alignment | Secure leadership endorsement and embed dashboard review into standing oversight meetings |
| Data Infrastructure | CTMS integration was essential | - Automated daily extracts enabled reliability - Historical data inconsistencies required remediation | Improved long‑term data quality | Conduct baseline data audits and plan for early data cleanup before launch |
| Data Quality Processes | Automated validation added value | - Early detection of missing/invalid fields - Initial increase in correction workload | Shifted QC upstream | Pair automated QC with clear ownership and response workflows |
| User Adoption | Role‑based design improved usability | - Minimal training required - Early skepticism around transparency | Sustained engagement across roles | Use role‑specific views and emphasize supportive—not punitive—use of data |
| Transparency & Culture | Increased visibility changed behavior | - Encouraged accountability - Initial resistance to comparative views | Cultural shift toward data‑driven discussions | Frame dashboards as improvement tools and socialize expectations early |
| Operational Decision‑Making | Forecasting supported proactive planning | - Enabled anticipatory interventions - Forecast uncertainty required contextual interpretation | Earlier identification of accrual risks | Pair forecasts with operational context and qualitative input from study teams |
| Maintenance & Sustainability | Continuous support was required | - Modular design enabled enhancements | Long‑term integration into workflows | Allocate dedicated informatics and analytics support for maintenance |

**Supplementary Table S2. Implementation lessons learned and recommendations for practice**
